# Obesity, Vascular Disease and Frailty in Aging Women with HIV

**DOI:** 10.20900/agmr20210014

**Published:** 2021-06-22

**Authors:** Deborah R. Gustafson, Samy I. McFarlane

**Affiliations:** 1 Section for NeuroEpidemiology, Department of Neurology, State University of New York Downstate Health Sciences University, Brooklyn, New York, NY 11203, USA; 2 University of Skövde, 541 28 Skövde, Sweden; 3 Division of Endocrinology, Department of Medicine, College of Medicine, State University of New York Downstate Health Sciences University, Brooklyn, New York, NY 11203, USA

**Keywords:** frailty, HIV, obesity, cardiovascular disease, aging, bone

## Abstract

Women with chronic HIV infection (WWH) living in the United States, experience a disproportionately high rate of obesity compared to uninfected populations. Both overweight and obesity, particularly central obesity, are major contributors to insulin resistance, hypertension, and dyslipidemia—the major components of metabolic syndromes, including type 2 diabetes, and leading to increased cardiovascular risk, including coronary heart disease, and cerebrovascular diseases. Notably, declining physical performance and frailty co-occur with vascular morbidities as well as changes in bone. These factors tend to exacerbate each other and accelerate the aging trajectory, leading to poorer quality of life, cognitive impairments, dementia, and eventually, death. In WWH, persistent HIV infection, sustained treatment for HIV infection, and concomitant obesity, may accelerate aging-related morbidities and poorer aging outcomes. Furthermore, health disparities factors common among some WWH, are independently associated with obesity and higher vascular risk. The purpose of this review is to describe the constellation of obesity, cardio- and cerebrovascular diseases, bone health and frailty among aging WWH, a 21st century emergence.

## INTRODUCTION

The HIV epidemic, in its fourth decade, is a treatable chronic condition affecting an increasingly older population [1]. Women with chronic HIV infection (WWH) experience disproportionate obesity and are living longer than ever observed in the history of the HIV epidemic. In the United States, the HIV epidemic began with the first reporting of AIDS in 1981 [2]. Over the last 40 years, advancements in HIV infection treatment regimens have led to a medical ‘miracle’—the successful treatment and control of HIV infection. Introduction of antiretroviral therapies (ART) and optimization of ART regimens have led to a growing population of older adults with chronic HIV infection never before observed in human history. However, more effective, newer generations of ART tend to promote body weight gain to obese levels (body mass index (BMI) ≥30 kg/m^2^), and increase cardiometabolic risk. Observational longitudinal cohorts of WWH report an average BMI that is obese as well as predominant central obesity (waist circumference >88 cm), which is a major risk factor for insulin resistance and cardiovascular disease (CVD) [3,4]. In addition, WWH continue to show lower bone mineral density (BMD) compared to uninfected women [5].

The HIV-aging phenomenon coincides with global aging. Vascular diseases, both cardio- and cerebrovascular diseases, were among the top 10 most common causes of death worldwide in 2019 [6]. Ischemic heart disease is the number one cause of death, followed by stroke (number two, Alzheimer’s disease (AD) (number 7, and Type 2 diabetes (number 9). AD is included among the top 10, since it is a neurodegenerative disease with a strong evidence base of vascular risk factors [7,8] and neurovascular etiology [9]. One of the reasons for vascular diseases comprising four of the top 10 causes of death is their association with the evolving pandemic of obesity. Approximately 39% of the world’s adult population was overweight and 13% were obese according to 2016 global estimates [10]. Obesity during middle-age is associated with higher risk of several adult life cardiovascular risk factors and events including hypertension, dyslipidaemia, type 2 diabetes (T2D), sleep disorders, atherosclerosis, and overall CVD [3,10]. These CVD risk factors are subsequently associated with cerebrovascular diseases such as stroke and late-onset, sporadic AD and related dementias (ADRD) [7], including vascular cognitive impairments and vascular dementias [11]. In addition, while higher BMI increases weight-bearing and enhances BMD, at obese levels and with the presence of other chronic conditions and limitations in mobility, this may not be the case, particularly among Black women [12].

**Figure 1.**
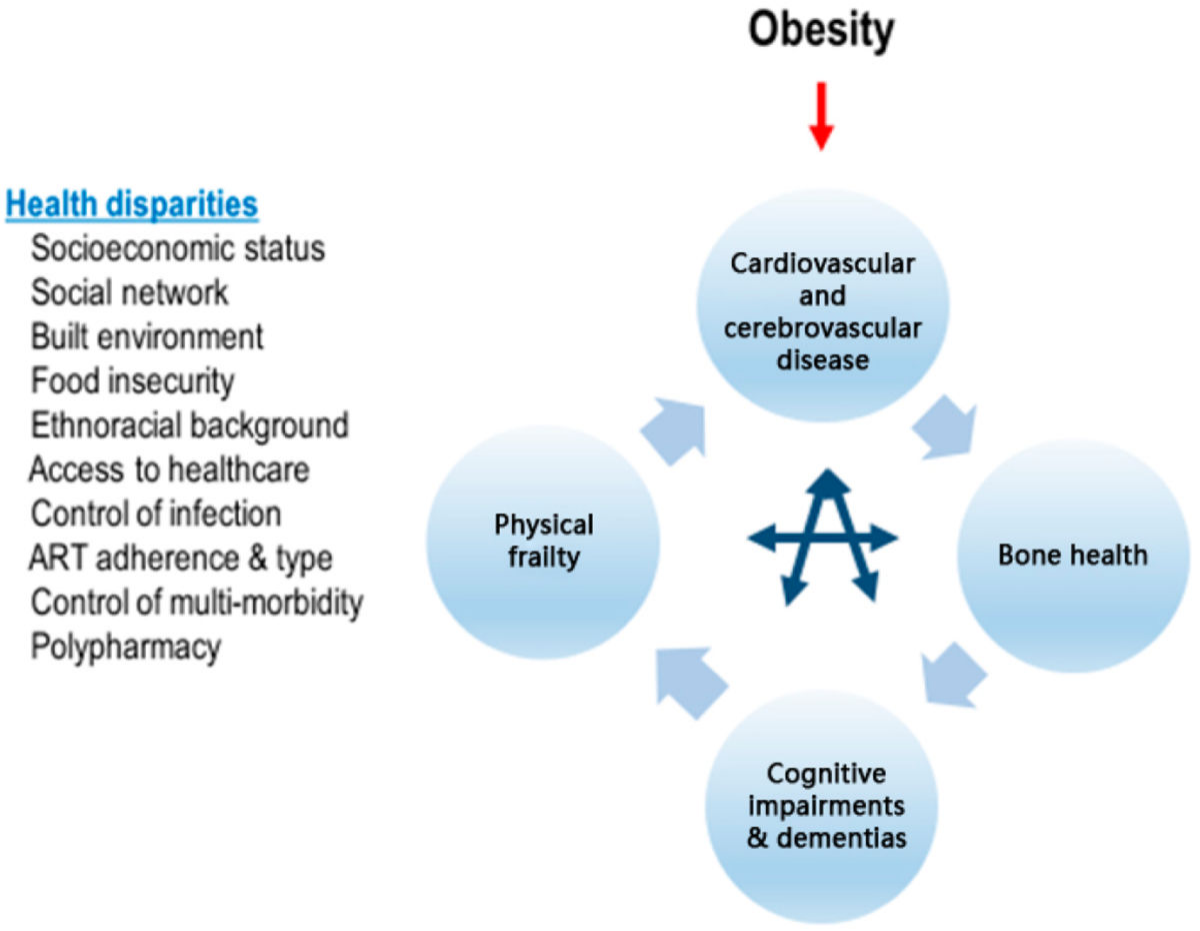
A constellation of obesity, vascular, bone, frailty and brain aging factors against a background of health disparities in aging women with chronic HIV infection.

Obesity among WWH is not only related to ART, but can also result from health disparities factors that are pervasive among aging WWH. These factors interfere with healthy living practices, and access to public health and health care interventions for obesity, T2D, CVD and cerebrovascular diseases [13–19]. Health disparities arise from variations in sociodemographic variables (age, income, education race/ethnicity and culture); challenging built environments that are common among major urban centers with high population densities and inherent barriers to physical activity promoting sedentary lifestyles; food insecurities; food deserts characteristic of urban environments (areas where access to healthy food is limited and unaffordable); employment and unemployment stresses; dealing with competing interests such as care-giving for children, grandchildren, aging parents, and other family members; and lack of social support. (See Figure 1) Thus, primary prevention of obesity is largely neglected, often followed by poor management of sequelae as secondary and tertiary intervention strategies, and bone health is often ignored.

The importance of multi-morbid HIV infection, obesity, vascular diseases and poor bone health is not only a serious issue for the medical community, but has been echoed in discussions among WWH [20]. A report of the HIV and Aging workgroup, convened by the NIH Office of AIDS Research, discussed the importance of engaging HIV cohorts, such as the Women’s Interagency HIV Study (WIHS) to address issues related to aging and HIV [21]. Longitudinal case-control studies such as the WIHS (now the MACS/WIHS Combined Cohort Study, 27 years old in 2021 and funded through 2026) provide a strong foundation for studying physical, cognitive and biological aging, in health disparities communities.

In this review, we address the obesity that is associated with HIV infection in aging women and its role in vascular diseases, bone health and frailty. Our hypothesis is that WWH experience more obesity, which leads to higher risk for vascular diseases; and that this is concomitant with poor bone health. Together, these factors influence aging frailty characterized by decline in physical functioning, with corresponding cognitive decline and dementia.

## OBESITY IN HIV INFECTION

Among WWH, ART must be considered in the evaluation of obesity, CVD risk and frailty. ART was introduced in 1996 and since then, control of HIV infection in geographical areas and health care venues with access to and proactive use of ART, has markedly improved. ART is the first-line of HIV treatment. Adherence to ART reduces HIV viral load to undetectable levels (<40–75 copies/mL) and increases CD4^+^ counts to desirable levels (>500 cells/mL). It is ideally prescribed within 24 h of HIV infection diagnosis in major urban medical centers serving high-risk populations. ART has increased the probability of longer-term survival that is almost equivalent to those without infection [22]. Despite the success of ART in controlling HIV infection, adherence to several ART increases total and central obesity [23,24], CVD and potentially cerebrovascular disease [25,26]. Integrase strand-transfer inhibitor (INSTI)-based ART in particular, is associated with severe body weight gain [24,27]. In the WIHS for example, over a 2 year average follow-up, women who switched to an INSTI-based ART or added it to their ongoing regimen experienced an average increase of 2.1 kg in body weight; 0.8 kg/m^2^ in BMI; 1.4% in percent body fat; and 2.0, 1.9, 0.6, and 1.0cm in waist, hip, arm, and thigh circumferences, respectively (all *p* < 0.05). There were no differences in magnitude of these changes by INSTI type [27].

Underweight, overweight and obesity are commonly defined anthropometrically among those with and without HIV infection. Measures include BMI, waist circumference (WC), and waist-to-hip ratio (WHR). Generally in adults, either total (based on BMI) or central (based on WC or WHR) obesity indicates higher amounts of adipose tissue *versus* other body tissue types. Table 1 summarizes the National Heart, Lung and Blood Institute (NHLBI) classifications of total and central obesity associated with CVD outcomes and mortality [28].

## FRAILTY IN HIV INFECTION

Frailty among HIV populations is associated with functional impairment and disability [29]. Published in 2003, the Fried Frailty Index, more accurately termed the Fried Frailty Phenotype (FFP), is a useful construct with which to predict poor quality of life, cognitive impairment, dementias and death [30], particularly in usual aging elderly (65 years and older) [31]. The FFP has been used in studies of HIV as well, and defined using well-described criteria [32]. Using the FFP, frailty is defined as exhibiting at least three of five characteristics: (1) impaired mobility, (2) reduced grip strength, (3) physical exhaustion, (4) unintentional weight loss and (5) low physical activity. In the WIHS, mobility was measured using a 4 meter timed gait test using cut-offs from the Cardiovascular Health Study or from similar, at-risk uninfected adults [32,33]. Grip strength was measured using a dominant hand-held dynamometer with maximum force, using cut-offs from the CHS or from similar, at risk uninfected adults. Physical exhaustion was measured as a “Yes” to the question: “During the past four weeks, as a result of your physical health, have you had difficulty performing your work or other activities (for example, it took extra efforts)”? Low physical activity was a “Yes” to “Does your health now limit you in vigorous activities, such as running, lifting heavy objects, or participating in strenuous sports?” Unintentional weight loss was a “Yes” to: “Since your last visit, have you had unintentional weight loss of at least 10 pounds?” Some of these latter three components can be verified to some extent by clinical measurements.

The FFP has been measured in middle-aged WWH who may be at risk for premature aging [29,34]. These studies show that these WWH experience a prevalence of the FFP often greater than that observed in elderly [33,35,36]. The reason for this early manifestation of the FFP may be a consequence of the HIV infection itself, including suboptimal medication and control of infection early on, comorbid diseases (infectious or non-infectious) [35,37] and/or lifestyle and health disparities factors that may be common among women with HIV infection, such as smoking and substance use and abuse [38].

## OBESITY AND FRAILTY

Several published reports suggest a U-shaped relationship between BMI and the FFP in uninfected elderly. This is similar to the well-published U-shaped relationship between BMI and mortality, where both the upper (overweight and obesity) and lower (underweight) ends of the BMI distribution are associated with higher mortality (See Table 1) [39]. Aging with obesity and frailty, sometimes referred to as ‘obesity frailty’ is induced by sarcopenia [40] or myosteatosis [41] that arises from the combination of obesity and changes in skeletal muscle. This may be especially relevant for WWH compared to men with HIV infection [42]. Obesity frailty accompanies and may precede aging-related physical frailty, which is also accompanied by aging-related changes in skeletal muscle and bone, as well as being associated with health disparities [43].

In WWH to date, a U-shaped relationship between BMI and FFP has not been replicated for two primary reasons. First, adults infected with HIV at the beginning of the epidemic were primarily men and died before the age of 50 years from Acquired Immunodeficiency Syndrome (AIDS) or AIDS-related illnesses and complications that were accompanied by extreme body weight loss and cachexia. Second, comparably aging WWH who are adherent to ART are scarce. Even so, it may be difficult to evaluate given the higher average BMI of these women. While the ability to analyze continuous BMI distributions as part of life and disease course natural histories is not universal, the benefit of long-term monitoring of this phenomenon remain.

At least one report from the WIHS suggested no multivariate-adjusted association between BMI and the FFP in WWH or at risk uninfected women at average age 38 years [33], despite the existence of a univariate association. In this same group of women, the FFP at middle age was predictive of death over approximately 8 years in survival models evaluating a combination of physical frailty (FFP), metabolic (Veterans Aging Cohort Study (VACS) Index) frailty, and mental health (Centers for Epidemiologic Studies-Depression (CES-D)) indices [44]. In all aforementioned analyses there was adjustment for sociodemographic, CVD and aging factors, and BMI.

## OBESITY, OSTEOPOROSIS AND CARDIOVASCULAR DISEASE

The association between obesity and CVD has been long-observed, with potential pathophysiological mechanisms based on vascular, inflammatory, endocrine, metabolic, and neurovascular dysregulation [4]. The strong role of obesity in CVD risk is evident based on the inclusion of BMI in diabetes and CVD risk scores such as the American Diabetes Association and Finnish Diabetes Risk Score (FINDRISC) [45], and Framingham CVD risk scores [46].

While seemingly unrelated disorders that increase with age, accumulating evidence indicate a common seed and soil hypothesis and shared pathogenetic mechanisms among obesity, CVD and osteoporosis [47–52]. In 100 obese African-American women (average age, 63 years; average BMI 26.6 kg/m^2^), age, hypertension, and low BMD were independent predictors of higher arterial stiffness. Thus, the bone mineral loss leading to lower BMD may increase CVD risk in African-Americans independent of obesogenic effects [49]. Underlying mechanisms postulated for this complex relationship of obesity, osteoporosis and CVD include dyslipidemia, oxidative stress, inflammation, hyperhomocysteinemia, hypertension, and diabetes, all of which have also been associated with higher risk of low BMD or increased bone fragility [48]. Furthermore, nitric oxide, known for its atheroprotective effects, plays a role in osteoblast function and bone turnover. In a randomized controlled trial, nitroglycerine (a nitric oxide donor) was as effective as estrogen in preventing bone loss in women with surgical menopause [53].

## OBESITY, OSTEOPOROSIS AND FRAILTY

Accumulating evidence indicates that both obesity and osteoporotic fractures are associated with faster frailty progression [54]. The Canadian Multicentre Osteoporosis Study (CaMos) identified risk factors for frailty progression among over 5500 community-dwelling women age 50 years and older. Rates of change in frailty over 10 years were examined using the 30-item CaMos Frailty Index. Both obesity and new vertebral or hip fractures were associated with frailty progression [54]. Sedentary lifestyle is a possible explanation for this observed association [55]; and/or obesity frailty, a sarcopenic condition.

However, as aforementioned, the relationship between osteoporosis and obesity is complex and obesity is often shown as protective against osteoporosis [56]. Postulated mechanisms for protection in postmenopausal obese women include increased adipose tissue aromatization of androstenedione, secreted from the adrenal glands, into bioactive estrone that maintains or increases bone mass [56,57]; greater weight-bearing concurrent with obesity [56,57]; and the decreased SHBG in obesity that is associated with higher circulating free sex steroids and hyperinsulinemia. This may lead to reduced production of IGFBG-1, an increase in IGF-1, and subsequent osteoblast proliferation and increased BMD [56]. In addition, serum leptin is strongly correlated with BMI, even in WWH (BMI × leptin, *r* > 0.70) [58]. Leptin may exert peripheral effects similar to estrogen, leading to osteoblastic differentiation and increases in BMD [57]. However, it is important to note that leptin has very complex actions on bone, including inhibition of bone formation through actions on the hypothalamus [59] as well as effects on bone resorption through regulation of neural pathways [60], therefore, it controls both aspects of bone remodeling.

Despite biological plausibility, there is inconsistent evidence for a positive association between high BMI and bone health. First, central obesity (higher WC or WHR) increases osteoporosis risk even in non-obese adults [61]. Furthermore, the protective effects of obesity on BMD that have been documented among Whites are not necessarily replicated in other race/ethnic groups [12]. In a study by our group, among over 3000 White and Black women (average age 58 years, average BMI 30.6 kg/m^2^), per 1.0 kg/m^2^ BMI, the odds of having a BMD one standard deviation below the average peak BMD, was less (OR = 0.90, 95% CI 0.87–0.94, *p* < 0.01) among White women, compared to Black women, among whom a higher odds was observed when compared to Whites (OR 1.015, 95% CI 1.01–1.14, *p* < 0.01). These data indicate a potential race-dependent association of obesity with BMD, perhaps due to genetic background, lower levels of physical activity, or lower circulating vitamin D levels among Black compared to White women [62].

Superimposed upon observations in uninfected women, is HIV infection status and ART adherence, which paints a more complex bone health picture [23]. In the WIHS, among WWH who were on average obese and adherent to ART, one report suggested that older age, White (vs Black) race, prior fracture, history of cocaine use, and history of injection drug use were significant predictors of incident fracture, a marker of low BMD, over 10 years follow-up [63]. However, when these women were evaluated over 5.4 years during premenopause only, there was no association of HIV status with fracture risk; and traditional risk factors such as White (vs Black) race, hepatitis C virus infection, and higher serum creatinine were associated with fracture [64]. In another report from the WIHS, among WWH (*N* = 246) and uninfected women (*N* = 219), average age 45 years and obese, non-Black women experienced greater total hip BMD loss, while Black women experienced greater menopause-associated decline in total hip BMD compared with non-Black women [63]. In addition, among 437 WWH and without infection [5] cross-sectional observations showed that WWH have degraded skeletal microarchitecture (27% vs 9%, *P* = 0.001) and a lower mean lumbar spine (LS) trabecular bone score (TBS) (1.3 ± 0.1 vs 1.4 ± 0.1, *P* < 0.001) than uninfected women, after adjusting for age, race, menopause status, and BMI. However, there were no differences between WWH and uninfected women in the correlation between annual change in TBS and LS BMD change; and mean percent annual change in TBS (−1.0%/year ± 2.9% for WWH vs −0.8%/year ± 1.7% for uninfected women, *P* = 0.42). A plausible explanation that annual changes in TBS and BMD do not vary significantly between infected and uninfected women is that while HIV itself causes bone loss, much of the ART-related bone loss occurs acutely, during the first 1–2 years [65].

Falls, another indicator of low BMD, were associated with the FFP in WWH, but not necessarily HIV status [66]. In the WIHS, the FFP predicted an almost 2-fold higher odds of recurrent, but not single, falls among middle-aged WWH. Of the FFP components, unintentional weight loss and exhaustion were most informative [67]. Overall, data suggest a multifaceted bone health and frailty risk profile among obese WWH.

## OBESITY AND THE BRAIN

Epidemiological studies show that higher midlife BMI and WC are associated with a higher risk of late-onset, sporadic dementias, including AD, which are typically diagnosed at age 65 years or older [68–73]. BMI and central adiposity have also been associated with cognitive impairments in adults aged ≥70 years [74]. Domain-specific associations are also observed. For example, longer exposure to elevated BMI and WC in midlife has been associated with lower memory function at 60–64 years of age [75]; and higher BMI and larger WC at midlife has been associated with lower executive function 10 years later [76].

There is a paucity of comparable reports among WWH, due to the younger age of surviving WWH, the rarity of studies that include cognitive and dementia outcomes, as well as adequate duration of longitudinal follow-up. However, in one multivariable-adjusted cross-sectional analyses, among women with or at-risk for HIV infection in the WIHS, both higher BMI and WC were associated with better cognitive performance at average age 38 years [77]. It was deemed that a higher BMI is a marker for ART adherence, and that these women possess a healthier risk profile.

The higher amounts of adipose tissue accompanying overweight and obesity may contribute to the alterations in peripheral and cerebral circulation that are more pronounced with aging [78]. Adipose tissue and its secretory products (adipokines, cytokines, sex hormones, free fatty acids), contribute to vascular and metabolic effects on human health, and influence function in numerous tissues including the brain [79]. Actions occur both peripherally and centrally, and include inducing atherosclerosis in large carotid and cerebral arteries, and impairing peripheral microvascular function and cerebral microcirculation.

Both cardio- and cerebrovascular disease play key roles in the development of cognitive impairments and dementia [9]. In the brain, outcomes include decreased cerebral blood flow and Cerebral Small Vessel Disease (CSVD). CSVD is comprised of white matter hyperintensities (WMH), lacunes, microbleeds, and neurodegeneration on brain MRI; and obesity has been associated with these CSVD components [80–82]. Alterations in the cerebral circulation may also contribute to Vascular Cognitive Impairments and AD given the essential role of maintaining cerebral blood flow and the neurovascular unit [9]. ‘Every neuron has a capillary’ to maintain normal neuronal function [83]. Practically, the Framingham Stroke Risk Profile (FSRP), while not including BMI in its algorithm, includes sequelae of obesity such as high systolic blood pressure and T2D [84]. Given that WWH present for the first time at older ages when late-life cognitive impairments and dementias occur, with lifetimes of exposure to high vascular and cardiometabolic risk, the threat of adverse brain events and vascular cognitive impairments with aging is high.

## A POTENTIAL GESTALT: STRESS LINKS OBESITY, VASCULAR DISEASES, FRAILTY, BONE AND COGNITION, IN AGING WOMEN WITH HIV INFECTION

Developmental origins hypotheses link brain and bone in earliest and latest life [85]. Small head circumference and low birth weight are associated with age-related cognitive dysfunction in uninfected individuals [86]; and lower birth weights (age-adjusted OR = 1.72; 95% CI 1.29–2.28, *P* < 0.001) and shorter gestational time (age-adjusted OR = 1.13; 95% CI 1.04–1.24, *P* = 0.005) are associated with a higher odds of lifetime depression [87]. In addition, there is evidence for both heart-brain and fatbrain axes [88,89]. Allostasis is a theoretical model suggesting that individual differences in susceptibility to stress over the life course, are linked to behavioral responses to environmental challenges, as well as developmental origins. These behaviors are coupled to physiologic and pathophysiologic responses [90], and there is a process whereby the body responds to stress to maintain homeostasis. Understanding long-term health of WWH requires a life course perspective.

This complex scenario baffles our abilities to tease apart health outcomes among WWH. Thus, the definition of allostasis is extended as the cost of chronic exposure to fluctuating or heightened neuroendocrine responses resulting from repeated or chronic environmental challenges and social burden that an individual reacts to as being particularly stressful [90,91]. Thus, the combination of aging and health disparities directly affect neural mechanisms contributing to cognition and cognitive decline, as well as frailty and depressive symptoms [92]. Considerations for WWH are that HIV infection plus obesity contribute a profound allostatic load comprised of external stressors evoked by health disparities, such as food insecurities and sedentary lifestyles; and internal stressors including enhanced vascular, metabolic and neuronal stress. Repeatedly high stress levels alter the biology of stress and appetite/energy regulation, with both components directly affecting neural mechanisms contributing to stress-induced and food cue-induced motivation that involves encouragement through signals (verbal and non-verbal) and engagement in overeating of foods that enhance risk of weight gain and obesity. While the ‘jolly fat’ hypothesis, which proposed that obesity correlated inversely with depressive symptoms, was first reported in 1976 [93], women in particular, are not observed to be ‘jolly fat’ [94]. However, in the WIHS, there was a null association between obesity and depressive symptom score cross-sectionally at average age 38 years [95]. Yet, for over 20 years, obesity-related stress has been pinpointed as a major factor associated with lack of well-being [96].

Finally, while we described factors related to frailty among HIV-infected women, further research is needed to define effective interventions to prevent and treat frailty among these vulnerable populations, apart from the traditional life style interventions (diet and exercise) that are deemed insufficient among populations with HIV disease [97].

## CONCLUSION

**Figure 2.**
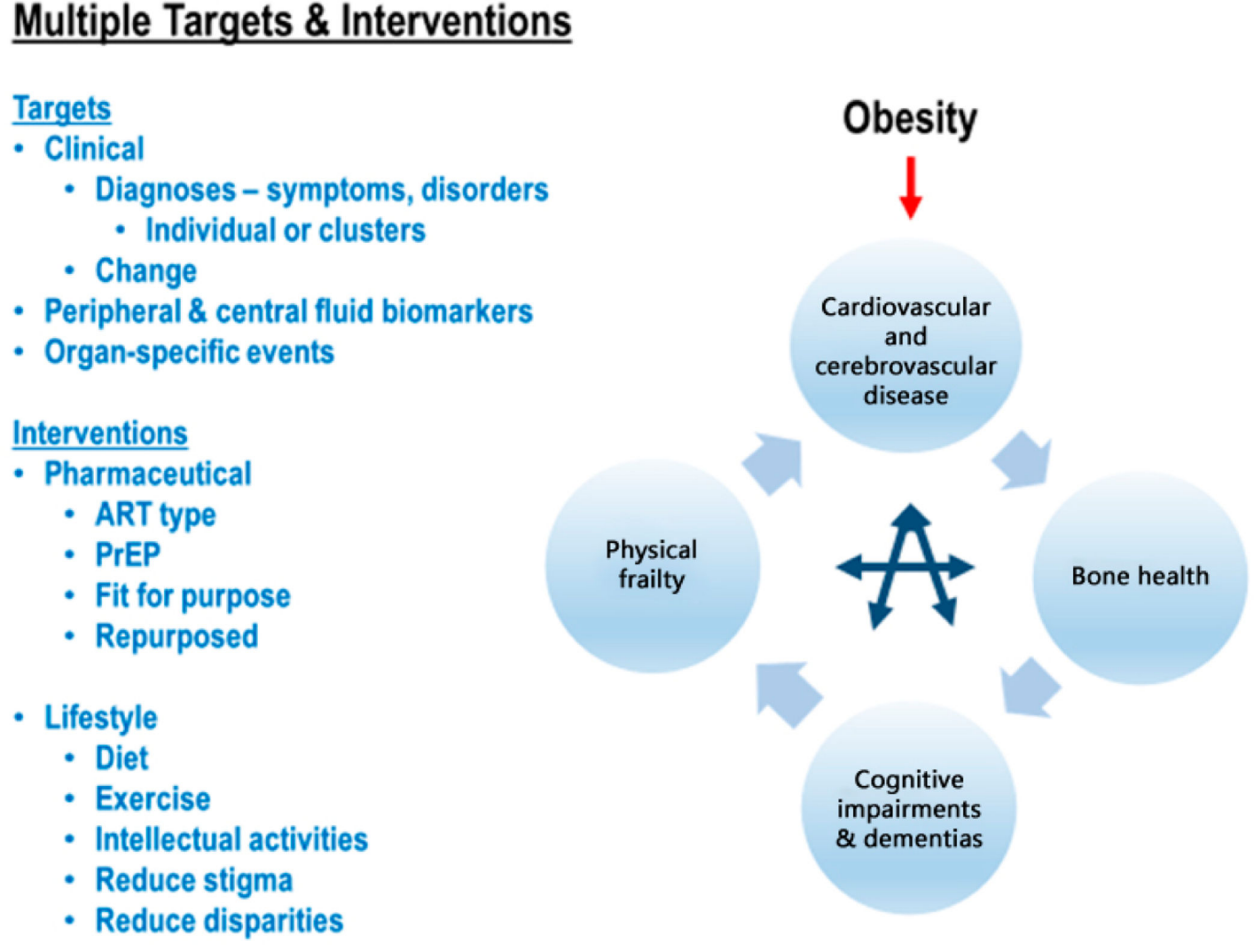
Multiple treatment targets and interventions in aging women with chronic HIV infection. ART, antiretroviral therapies; PrEP, Pre-exposure prophylaxis.

A review of a well-established literature evidences relationships among obesity, CVD, and bone health in physical frailty and brain aging among WWH. When considered against a background of health disparities, there may manifest prevention approaches not considered previously, as illustrated in Figure 2.

It is unclear whether WWH and obesity experience accelerated and/or aging with excess morbidities, and whether more adverse aging is related to the infection per se, the inflammatory and metabolic effects of the infection and adipose tissue, obesity effects of HIV medications, or an overall HIV + obesity-initiated and/or -mediated increase in allostatic load over the life course [98,99]. Better understanding of this area will propel us forward to a new and novel area of investigation linking brain and periphery in aging HIV [73,79].

## Figures and Tables

**Table 1.** Classification of overweight and obesity by BMI and WC, and disease ^1^ risk for women [28].

	Disease Risk Relative to Normal BMI and WC ^2^			
	BMI (kg/m^2^)	Obesity class	Women ≤ 88 cm (35 in)	Women > 88 cm (35 in) ^3^
**Underweight**	<18.5			
**Normal**	18.5–24.9			
**Overweight**	25.0–29.9		Increased	High
**Obesity**	30.0–34.9	1	High	Very high
	35.0–39.9	2	Very high	Very High
**Extreme Obesity**	≥40	3	Extremely high	Extremely high

1 Disease risk for type 2 diabetes, hypertension, and CVD.

2 Waist Circumference.

3 Increased WC is a marker for increased risk, even in women of normal weight.

## ABBREVIATIONS

WWH: women with HIV infection
HIV: human immunodeficiency virus
ART: antiretroviral therapies
kg: kilograms
m: meter
BMI: body mass index
CVD: cardiovascular disease
BMD: bone mineral density
AD: Alzheimer’s disease
T2D: type 2 diabetes
ADRD: AD and related dementias
WIHS: Women’s Interagency HIV Study
ml: milliliter
INSTI: integrase strand-transfer inhibitor
cm: centimeter
WC: waist circumference
WHR: waist-to-hip ratio
NHLBI: National Heart, Lung and Blood Institute
FFP: Fried Frailty Phenotype
AIDS: Acquired Immunodeficiency Syndrome
VACS: Veterans Aging Cohort Study
CES-D: Centers for Epidemiologic Studies—Depression
FINDRISC: Finnish Diabetes Risk Score
CaMos: Canadian Multicentre Osteoporosis Study
IGFBG-1: Insulin Like Growth Factor Binding Protein
IGF-1: Insulin Like Growth Factor-1
r: correlation coefficient
OR: odds ratio
95% CI: 95% Confidence Interval
TBS: trabecular bone score
%: percent
CSVD: Cerebral Small Vessel Disease
WMH: white matter hyperintensities
MRI: magnetic resonance imaging
FSRP: Framingham Stroke Risk Profile
PrEP: Pre-exposure prophylaxis

## References

[1] UNAIDS. UNAIDS Report on the Global AIDS Epidemic 2010. Geneva (Switzerland): UNAIDS; 2010. Report No.: UNAIDS/10.11E | JC1958E.

[2] Centers for Disease Control and Prevention. HIV and AIDS—United States, 1981–2000. Available from: https://www.cdc.gov/mmwr/preview/mmwrhtml/mm5021a2.htm. Accessed 2021 Jun 16.

[3] Classification of Overweight and Obesity by BMI, Waist Circumference, and Associated Disease Risks. Available from: https://pro.aace.com/files/obesity/toolkit/classification_of_obesity_and_risks.pdf. Accessed 2021 Jun 16.

[4] McFarlane SI, Banerji M, Sowers JR. Insulin resistance and cardiovascular disease. J Clin Endocrinol Metab. 2001;86(2):713–8. 11158035 10.1210/jcem.86.2.7202

[5] Sharma A, Ma Y, Tien PC, Scherzer R, Anastos K, Cohen MH, HIV Infection Is Associated With Abnormal Bone Microarchitecture: Measurement of Trabecular Bone Score in the Womenʼs Interagency HIV Study. J Acquir Immune Defic Syndr. 2018;78(4):441–9. 29940603 10.1097/QAI.0000000000001692 PMC6020168

[6] WHO. Top 10 Causes of Death. World Health Organization. Geneva (Switzerland): World Health Organization; 2020.

[7] Fitzpatrick AL, Kuller LH, Lopez OL, Diehr P, OʼMeara ES, Longstreth WT Jr, Midlife and late-life obesity and the risk of dementia: cardiovascular health study. Arch Neurol. 2009;66(3):336–42. 19273752 10.1001/archneurol.2008.582 PMC3513375

[8] Exalto LG, Quesenberry CP, Barnes D, Kivipelto M, Biessels GJ, Whitmer RA. Midlife risk score for the prediction of dementia four decades later. Alzheimers Dement. 2014;10(5):562–70. 24035147 10.1016/j.jalz.2013.05.1772

[9] Zlokovic BV. Neurovascular pathways to neurodegeneration in Alzheimerʼs disease and other disorders. Nat Rev Neurosci. 2011;12(12):723–38. 22048062 10.1038/nrn3114 PMC4036520

[10] WHO. Obesity and Overweight. Geneva (Switzerland): World Health Organization; 2020.

[11] Rosenberg GA. Vascular cognitive impairment: Biomarkers in diagnosis and molecular targets in therapy. J Cereb Blood Flow Metab. 2016;36(1):4–5. 26667984 10.1177/0271678X15609542 PMC4758564

[12] Castro JP, Joseph LA, Shin JJ, Arora SK, Nicasio J, Shatzkes J, Differential effect of obesity on bone mineral density in White, Hispanic and African American women: a cross sectional study. Nutr Metab (Lond). 2005;2(1):9. 15817133 10.1186/1743-7075-2-9 PMC1090614

[13] Babulal GM, Quiroz YT, Albensi BC, Arenaza-Urquijo E, Astell AJ, Babiloni C, Perspectives on ethnic and racial disparities in Alzheimer’s disease and related dementias: Update and areas of immediate need. Alzheimers Dement. 2019;15(2):292–312. 30555031 10.1016/j.jalz.2018.09.009 PMC6368893

[14] Belgrave FZ, Abrams JA. Reducing disparities and achieving equity in African American womenʼs health. Am Psychol. 2016;71(8):723–33. 27977253 10.1037/amp0000081

[15] Dicent Taillepierre JC, Liburd L, OʼConnor A, Valentine J, Bouye K, McCree DH, Toward Achieving Health Equity: Emerging Evidence and Program Practice. J Public Health Manag Pract. 2016;22(Suppl 1):S43–9. 26599028 10.1097/PHH.0000000000000375

[16] Golovaty I, Tien PC, Price JC, Sheira L, Seligman H, Weiser SD. Food Insecurity May Be an Independent Risk Factor Associated with Nonalcoholic Fatty Liver Disease among Low-Income Adults in the United States. J Nutr. 2020;150(1):91–8. 31504710 10.1093/jn/nxz212 PMC6946902

[17] Isaacs D, Riley AC, Prasad-Reddy L, Castner R, Fields H, Harper-Brown D, Jazzin’ Healthy: Interdisciplinary Health Outreach Events Focused on Disease Prevention and Health Promotion. J Racial Ethn Health Disparities. 2017;4(2):223–32. 27059051 10.1007/s40615-016-0221-4

[18] Rashid JR, Leath BA, Truman BI, Atkinson DD, Gary LC, Manian N. Translating Comparative Effectiveness Research Into Practice: Effects of Interventions on Lifestyle, Medication Adherence, and Self-care for Type 2 Diabetes, Hypertension, and Obesity Among Black, Hispanic, and Asian Residents of Chicago and Houston, 2010 to 2013. J Public Health Manag Pract. 2017;23(5):468–76. 28257397 10.1097/PHH.0000000000000525

[19] Singh GK, Daus GP, Allender M, Ramey CT, Martin EK, Perry C, Social Determinants of Health in the United States: Addressing Major Health Inequality Trends for the Nation, 1935–2016. Int J MCH AIDS. 2017;6(2):139–64. 29367890 10.21106/ijma.236 PMC5777389

[20] Gustafson DR. Conversation with Womenʼs Interagency HIV Study National Community Advisory Board. 2017 6 20.

[21] High KP, Brennan-Ing M, Clifford DB, Cohen MH, Currier J, Deeks SG, HIV and aging: state of knowledge and areas of critical need for research. A report to the NIH Office of AIDS Research by the HIV and Aging Working Group. J Acquir Immune Defic Syndr. 2012;60(Suppl 1):S1–18. 22688010 10.1097/QAI.0b013e31825a3668 PMC3413877

[22] Marcus JL, Leyden WA, Alexeeff SE, Anderson AN, Hechter RC, Hu H, Comparison of Overall and Comorbidity-Free Life Expectancy Between Insured Adults With and Without HIV Infection, 2000–2016. JAMA Netw Open. 2020;3(6):e207954. 10.1001/jamanetworkopen.2020.7954 PMC7296391 32539152

[23] Justman JE, Hoover DR, Shi Q, Tan T, Anastos K, Tien PC, Longitudinal anthropometric patterns among HIV-infected and HIV-uninfected women. J Acquir Immune Defic Syndr. 2008;47(3):312–9. 18197125 10.1097/QAI.0b013e318162f597 PMC4406344

[24] Lahiri CD, Xu Y, Wang K, Alvarez JA, Sheth AN, OʼHalloran J, Weight and Body Mass Index Change After Switching to Integrase Inhibitors or Tenofovir Alafenamide Among Women Living with HIV. AIDS Res Hum Retroviruses. 2021 6;37(6):461–7. 33231474 10.1089/aid.2020.0197 PMC8213005

[25] Palella FJ Jr, Phair JP. Cardiovascular disease in HIV infection. Current opinion in HIV and AIDS. 2011;6(4):266–71. 21546831 10.1097/COH.0b013e328347876c PMC3501268

[26] Summers NA, Lahiri CD, Angert CD, Aldredge A, Mehta CC, Ofotokun I, Metabolic Changes Associated With the Use of Integrase Strand Transfer Inhibitors Among Virally Controlled Women. J Acquir Immune Defic Syndr. 2020;85(3):355–62. 33060420 10.1097/QAI.0000000000002447 PMC7577246

[27] Kerchberger AM, Sheth AN, Angert CD, Mehta CC, Summers NA, Ofotokun I, Weight Gain Associated with Integrase Stand Transfer Inhibitor Use in Women. Clin Infect Dis. 2020 7 27;71(3):593–600. 31504324 10.1093/cid/ciz853 PMC7384314

[28] NHLBI Expert Panel. The Practical Guide. Identification, Evaluation, and Treatment of Overweight and Obesity in Adults. Bethesda: National Heart, Lung, and Blood Institute, North American Association for the Study of Obesity, 2000 October 2000. Bethesda (US): National Institutes of Health; 2000. Report No.: 00–4084.

[29] Erlandson KM, Schrack JA, Jankowski CM, Brown TT, Campbell TB. Functional impairment, disability, and frailty in adults aging with HIV-infection. Curr HIV/AIDS Rep. 2014;11(3):279–90. 24966138 10.1007/s11904-014-0215-y PMC4125474

[30] Hirsch C, Anderson ML, Newman A, Kop W, Jackson S, Gottdiener J, The association of race with frailty: the cardiovascular health study. Ann Epidemiol. 2006;16(7):545–53. 16388967 10.1016/j.annepidem.2005.10.003

[31] Shamliyan T, Talley KM, Ramakrishnan R, Kane RL. Association of frailty with survival: a systematic literature review. Ageing Res Rev. 2013;12(2):719–36. 22426304 10.1016/j.arr.2012.03.001

[32] Fried LP, Tangen CM, Walston J, Newman AB, Hirsch C, Gottdiener J, Frailty in older adults: evidence for a phenotype. J Gerontol A. 2001;56(3):M146–56. 10.1093/gerona/56.3.m146 11253156

[33] Gustafson DR, Shi Q, Thurn M, Holman S, Minkoff H, Cohen M, Frailty and Constellations of Factors in Aging HIV-infected and Uninfected Women--The Womenʼs Interagency HIV Study. J Frailty Aging. 2016;5(1):43–8. 26980368 10.14283/jfa.2016.79 PMC4957016

[34] Escota GV, Patel P, Brooks JT, Bush T, Conley L, Baker J, Short communication: The Veterans Aging Cohort Study Index is an effective tool to assess baseline frailty status in a contemporary cohort of HIV-infected persons. AIDS Res Hum Retroviruses. 2015;31(3):313–7. 25495766 10.1089/AID.2014.0225

[35] Cohen MH, Hotton AL, Hershow RC, Levine A, Bacchetti P, Golub ET, Gender-related risk factors improve mortality predictive ability of VACS Index among HIV-infected women. J Acquir Immune Defic Syndr. 2015 12 15;70(5):538–44. 26284531 10.1097/QAI.0000000000000795 PMC4644433

[36] Terzian AS, Holman S, Nathwani N, Robison E, Weber K, Young M, Factors associated with preclinical disability and frailty among HIV-infected and HIV-uninfected women in the era of cART. J Womens Health. 2009;18(12):1965–74. 10.1089/jwh.2008.1090 PMC2828186 20044858

[37] Verucchi G, Calza L, Manfredi R, Chiodo F. Human immunodeficiency virus and hepatitis C virus coinfection: epidemiology, natural history, therapeutic options and clinical management. Infection. 2004;32(1):33–46. 15007741 10.1007/s15010-004-3063-7

[38] Piggott DA, Muzaale AD, Mehta SH, Brown TT, Patel KV, Leng SX, Frailty, HIV infection, and mortality in an aging cohort of injection drug users. PLoS One. 2013;8(1):e54910. 10.1371/journal.pone.0054910 PMC3561408 23382997

[39] Weitoft GR, Eliasson M, Rosen M. Underweight, overweight and obesity as risk factors for mortality and hospitalization. Scand J Public Health. 2008;36(2):169–76. 18519281 10.1177/1403494807085080

[40] Hamrick MW, McGee-Lawrence ME, Frechette DM. Fatty Infiltration of Skeletal Muscle: Mechanisms and Comparisons with Bone Marrow Adiposity. Front Endocrinol. 2016;7:69. 10.3389/fendo.2016.00069 PMC4913107 27379021

[41] Miljkovic I, Zmuda JM. Epidemiology of myosteatosis. Curr Opin Clin Nutr Metab Care. 2010;13(3):260–4. 20179586 10.1097/MCO.0b013e328337d826 PMC2872135

[42] Echeverria P, Bonjoch A, Puig J, Estany C, Ornelas A, Clotet B, High Prevalence of Sarcopenia in HIV-Infected Individuals. Biomed Res Int. 2018;2018:5074923. 10.1155/2018/5074923 PMC6077654 30112397

[43] Mendham AE, Goedecke JH, Micklesfield LK, Brooks NE, Faber M, Christensen DL, Understanding factors associated with sarcopenic obesity in older African women from a low-income setting: a cross-sectional analysis. BMC Geriatr. 2021;21(1):247. 33853546 10.1186/s12877-021-02132-x PMC8048063

[44] Gustafson DR, Shi Q, Holman S, Minkoff H, Cohen MH, Plankey MW, Predicting death over 8 years in a prospective cohort of HIV-infected women: the Women’s Interagency HIV Study. BMJ Open. 2017;7(6):e013993. 10.1136/bmjopen-2016-013993 PMC5577878 28667199

[45] Lindstrom J, Tuomilehto J. The diabetes risk score: a practical tool to predict type 2 diabetes risk. Diabetes Care. 2003;26(3):725–31. 12610029 10.2337/diacare.26.3.725

[46] DʼAgostino RB Sr, Vasan RS, Pencina MJ, Wolf PA, Cobain M, Massaro JM, General cardiovascular risk profile for use in primary care: the Framingham Heart Study. Circulation. 2008;117(6):743–53. 18212285 10.1161/CIRCULATIONAHA.107.699579

[47] McFarlane SI. Bone metabolism and the cardiometabolic syndrome: pathophysiologic insights. J Cardiometab Syndr. 2006;1(1):53–7. 17675897 10.1111/j.0197-3118.2006.05457.x

[48] McFarlane SI, Muniyappa R, Shin JJ, Bahtiyar G, Sowers JR. Osteoporosis and cardiovascular disease: brittle bones and boned arteries, is there a link? Endocrine. 2004;23(1):1–10. 15034190 10.1385/ENDO:23:1:01

[49] McFarlane SI, Qureshi G, Singh G, Venner-Jones K, Salciccioli L, Lazar J. Bone Mineral Density as a Predictor of Atherosclerosis and Arterial Wall Stiffness in Obese African-American Women. Cardiorenal Med. 2012;2(4):328–34. 23381741 10.1159/000345461 PMC3551407

[50] Lampropoulos CE, Papaioannou I, DʼCruz DP. Osteoporosis—a risk factor for cardiovascular disease? Nat Rev Rheumatol. 2012;8(10):587–98. 22890244 10.1038/nrrheum.2012.120

[51] Laroche M, Pecourneau V, Blain H, Breuil V, Chapurlat R, Cortet B, Osteoporosis and ischemic cardiovascular disease. Joint Bone Spine. 2017;84(4):427–32. 27838246 10.1016/j.jbspin.2016.09.022

[52] Lian XL, Zhang YP, Li X, Jing LD, Cairang ZM, Gou JQ. Exploration on the relationship between the elderly osteoporosis and cardiovascular disease risk factors. Eur Rev Med Pharmacol Sci. 2017;21(19):4386–90. 29077156

[53] Wimalawansa SJ. Nitroglycerin therapy is as efficacious as standard estrogen replacement therapy (Premarin) in prevention of oophorectomy-induced bone loss: a human pilot clinical study. J Bone Miner Res. 2000;15(11):2240–4. 11092405 10.1359/jbmr.2000.15.11.2240

[54] Gajic-Veljanoski O, Papaioannou A, Kennedy C, Ioannidis G, Berger C, Wong AKO, Osteoporotic fractures and obesity affect frailty progression: a longitudinal analysis of the Canadian multicentre osteoporosis study. BMC Geriatr. 2018;18(1):4. 29304836 10.1186/s12877-017-0692-0 PMC5756402

[55] McPhee JS, French DP, Jackson D, Nazroo J, Pendleton N, Degens H. Physical activity in older age: perspectives for healthy ageing and frailty. Biogerontology. 2016;17(3):567–80. 26936444 10.1007/s10522-016-9641-0 PMC4889622

[56] Albala C, Yanez M, Devoto E, Sostin C, Zeballos L, Santos JL. Obesity as a protective factor for postmenopausal osteoporosis. Int J Obes Relat Metab Disord. 1996;20(11):1027–32. 8923160

[57] Crepaldi G, Romanato G, Tonin P, Maggi S. Osteoporosis and body composition. J Endocrinol Invest. 2007;30(6 Suppl):42–7. 17721073

[58] Gustafson DR, Mielke MM, Keating SA, Holman S, Minkoff H, Crystal HA. Leptin, Adiponectin and Cognition in Middle-aged HIV-infected and Uninfected Women. The Brooklyn Womenʼs Interagency HIV Study. J Gerontol Geriatr Res. 2015;4(5):240. 27536467 10.4172/2167-7182.1000240 PMC4984413

[59] Ducy P, Amling M, Takeda S, Priemel M, Schilling AF, Beil FT, Leptin inhibits bone formation through a hypothalamic relay: a central control of bone mass. Cell. 2000;100(2):197–207. 10660043 10.1016/s0092-8674(00)81558-5

[60] Elefteriou F, Ahn JD, Takeda S, Starbuck M, Yang X, Liu X, Leptin regulation of bone resorption by the sympathetic nervous system and CART. Nature. 2005;434(7032):514–20. 15724149 10.1038/nature03398

[61] Blaauw R, Albertse EC, Hough S. Body fat distribution as a risk factor for osteoporosis. S Afr Med J. 1996;86(9):1081–4. 8888774

[62] Coney P, Demers LM, Dodson WC, Kunselman AR, Ladson G, Legro RS. Determination of vitamin D in relation to body mass index and race in a defined population of black and white women. Int J Gynaecol Obstet. 2012;119(1):21–5. 22818533 10.1016/j.ijgo.2012.05.024 PMC3438362

[63] Sharma A, Flom PL, Rosen CJ, Schoenbaum EE. Racial differences in bone loss and relation to menopause among HIV-infected and uninfected women. Bone. 2015;77:24–30. 25896953 10.1016/j.bone.2015.04.018 PMC4418198

[64] Yin MT, Lu D, Cremers S, Tien PC, Cohen MH, Shi Q, Short-term bone loss in HIV-infected premenopausal women. J Acquir Immune Defic Syndr. 2010;53(2):202–8. 19890216 10.1097/QAI.0b013e3181bf6471 PMC2813405

[65] Arora S, Agrawal M, Sun L, Duffoo F, Zaidi M, Iqbal J. HIV and bone loss. Curr Osteoporos Rep. 2010;8(4):219–26. 20830538 10.1007/s11914-010-0036-x

[66] Sharma A, Hoover DR, Shi Q, Holman S, Plankey MW, Wheeler AL, Falls among middle-aged women in the Women’s Interagency HIV Study. Antivir Ther. 2016;21(8):697–706. 27427794 10.3851/IMP3070 PMC5243157

[67] Sharma A, Hoover DR, Shi Q, Gustafson DR, Plankey MW, Tien PC, Frailty as a predictor of falls in HIV-infected and uninfected women. Antivir Ther. 2019;24(1):51–61. 30604692 10.3851/IMP3286 PMC10141570

[68] Gustafson DR, Rothenberg E, Blennow K, Steen B, Skoog I. An 18-year follow up of overweight and risk for Alzheimerʼs disease. Arch Intern Med. 2003;163:1524–8. 12860573 10.1001/archinte.163.13.1524

[69] Anstey KJ, Cherbuin N, Budge M, Young J. Body mass index in midlife and late-life as a risk factor for dementia: a meta-analysis of prospective studies. Obes Rev. 2011;12(5):e426–37. 21348917 10.1111/j.1467-789X.2010.00825.x

[70] Whitmer RA, Gunderson EP, Quesenberry CP Jr, Zhou J, Yaffe K. Body mass index in midlife and risk of Alzheimer disease and vascular dementia. Curr Alzheimer Res. 2007;4(2):103–9. 17430231 10.2174/156720507780362047

[71] Whitmer RA, Gustafson DR, Barrett-Connor E, Haan MN, Gunderson EP, Yaffe K. Central obesity and increased risk of dementia more than three decades later. Neurology. 2008;71(14):1057–64. 18367704 10.1212/01.wnl.0000306313.89165.ef

[72] Tolppanen AM, Ngandu T, Kareholt I, Laatikainen T, Rusanen M, Soininen H, Midlife and late-life body mass index and late-life dementia: results from a prospective population-based cohort. J Alzheimers Dis. 2014;38(1):201–9. 23948937 10.3233/JAD-130698

[73] Emmerzaal TL, Kiliaan AJ, Gustafson DR. 2003–2013: a decade of body mass index, Alzheimer’s disease, and dementia. J Alzheimers Dis. 2015;43(3):739–55. 25147111 10.3233/JAD-141086

[74] Liu Z, Yang H, Chen S, Cai J, Huang Z. The association between body mass index, waist circumference, waist-hip ratio and cognitive disorder in older adults. J Public Health. 2019;41(2):305–12. 10.1093/pubmed/fdy121 30020483

[75] Masi S, Georgiopoulos G, Khan T, Johnson W, Wong A, Charakida M, Patterns of adiposity, vascular phenotypes and cognitive function in the 1946 British Birth Cohort. BMC Med. 2018;16(1):75. 29804545 10.1186/s12916-018-1059-x PMC5971427

[76] Kesse-GuyotE, AndreevaVA, TouvierM, JeandelC, FerryM, HercbergS, Overall and abdominal adiposity in midlife and subsequent cognitive function. J Nutr Health Aging. 2015;19(2):183–9.2565144410.1007/s12603-014-0508-2

[77] Gustafson DR, Mielke MM, Tien PC, Valcour V, Cohen M, Anastos K, Anthropometric measures and cognition in middle-aged HIV-infected and uninfected women. The Women’s Interagency HIV Study. J Neurovirol. 2013;19(6):574–85. 24338243 10.1007/s13365-013-0219-1 PMC3957488

[78] Tucsek Z, Toth P, Sosnowska D, Gautam T, Mitschelen M, Koller A, Obesity in aging exacerbates blood-brain barrier disruption, neuroinflammation, and oxidative stress in the mouse hippocampus: effects on expression of genes involved in beta-amyloid generation and Alzheimerʼs disease. J Gerontol A. 2014;69(10):1212–26. 10.1093/gerona/glt177 PMC4172034 24269929

[79] Arnoldussen IA, Kiliaan AJ, Gustafson DR. Obesity and dementia: adipokines interact with the brain. Eur Neuropsychopharmacol. 2014;24(12):1982–99. 24704273 10.1016/j.euroneuro.2014.03.002 PMC4169761

[80] Arnoldussen IAC, Gustafson DR, Leijsen EMC, de Leeuw FE, Kiliaan AJ. Adiposity is related to cerebrovascular and brain volumetry outcomes in the RUN DMC study. Neurology. 2019;93(9):e864–78. 31363056 10.1212/WNL.0000000000008002

[81] Gustafson D, Lissner L, Bengtsson C, Björkelund C, Skoog I. A 24-year follow-up of body mass index and cerebral atrophy. Neurology. 2004;63:1876–81. 15557505 10.1212/01.wnl.0000141850.47773.5f

[82] Gustafson D, Steen B, Skoog I. Body mass index and white matter lesions in elderly women. An 18-year longitudinal study. Int J Psychogeriatr. 2004;16:327–36. 10.1017/s1041610204000353 15559756

[83] Zlokovic BV. Neurodegeneration and the neurovascular unit. Nat Med. 2010;16(12):1370–1. 21135839 10.1038/nm1210-1370

[84] Singer J, Gustafson D, Cummings C, Egelko A, Mlabasati J, Conigliaro A, Independent ischemic stroke risk factors in older Americans: a systematic review. Aging. 2019;11(10):3392–407. 31127075 10.18632/aging.101987 PMC6555455

[85] Gluckman PD, Hanson MA, Pinal C. The developmental origins of adult disease. Matern Child Nutr. 2005;1(3):130–41. 16881892 10.1111/j.1740-8709.2005.00020.x PMC6860944

[86] Mosing MA, Lundholm C, Cnattingius S, Gatz M, Pedersen NL. Associations between birth characteristics and age-related cognitive impairment and dementia: A registry-based cohort study. PLoS Med. 2018;15(7):e1002609. 10.1371/journal.pmed.1002609 PMC6051563 30020924

[87] Gudmundsson P, Andersson S, Gustafson D, Waern M, Ostling S, Hallstrom T, Depression in Swedish women: relationship to factors at birth. Eur J Epidemiol. 2011;26(1):55–60. 20857177 10.1007/s10654-010-9508-7

[88] Tahsili-Fahadan P, Geocadin RG. Heart-Brain Axis: Effects of Neurologic Injury on Cardiovascular Function. Circ Res. 2017;120(3):559–72. 28154104 10.1161/CIRCRESAHA.116.308446

[89] Elmquist JK, Flier JS. Neuroscience. The fat-brain axis enters a new dimension. Science. 2004;304(5667):63–4. 15064411 10.1126/science.1096746

[90] McEwen BS. Stress, adaptation, and disease. Allostasis and allostatic load. Ann N Y Acad Sci. 1998;840:33–44. 9629234 10.1111/j.1749-6632.1998.tb09546.x

[91] Ludwig J, Sanbonmatsu L, Gennetian L, Adam E, Duncan GJ, Katz LF, Neighborhoods, obesity, and diabetes—a randomized social experiment. N Engl J Med. 2011;365(16):1509–19. 22010917 10.1056/NEJMsa1103216 PMC3410541

[92] Sinha R, Jastreboff AM. Stress as a common risk factor for obesity and addiction. Biol Psychiatry. 2013;73(9):827–35. 23541000 10.1016/j.biopsych.2013.01.032 PMC3658316

[93] Crisp AH, McGuiness B. Jolly fat: relation between obesity and psychoneurosis in general population. Br Med J. 1976;1(6000):7–9. 1247732 10.1136/bmj.1.6000.7 PMC1638245

[94] Palinkas LA, Wingard DL, Barrett-Connor E. Depressive symptoms in overweight and obese older adults: a test of the “jolly fat” hypothesis. J Psychosom Res. 1996;40(1):59–66. 8730645 10.1016/0022-3999(95)00542-0

[95] Groysman AY, Keating S, Holman S, Weedon J, Minkoff H, Gustafson DR. Depression, leptin, adiponectin, and obesity in women with and without HIV infection. The Womenʼs Interagency HIV Study. Neurol Neurobiol. 2018;1:2–9.

[96] Crocker J, Cornwell B, Major B. The stigma of overweight: affective consequences of attributional ambiguity. J Pers Soc Psychol. 1993;64(1):60–70. 8421252 10.1037//0022-3514.64.1.60

[97] Erlandson KM, Piggott DA. Frailty and HIV: Moving from Characterization to Intervention. Curr HIV/AIDS Rep. 2021;18(3):157–75. 33817767 10.1007/s11904-021-00554-1 PMC8193917

[98] Nguyen N, Holodniy M. HIV infection in the elderly. Clin Interv Aging. 2008;3(3):453–72. 18982916 10.2147/cia.s2086 PMC2682378

[99] Kalayjian RC, Landay A, Pollard RB, Taub DD, Gross BH, Francis IR, Age-related immune dysfunction in health and in human immunodeficiency virus (HIV) disease: association of age and HIV infection with naive CD8^+^ cell depletion, reduced expression of CD28 on CD8^+^ cells, and reduced thymic volumes. J Infect Dis. 2003;187(12):1924–33. 12792869 10.1086/375372

